# Neuroscientific and Genetic Evidence in Criminal Cases: A Double-Edged Sword in Germany but Not in the United States?

**DOI:** 10.3389/fpsyg.2019.02343

**Published:** 2019-10-16

**Authors:** Daniela Guillen Gonzalez, Merlin Bittlinger, Susanne Erk, Sabine Müller

**Affiliations:** ^1^Research Division of Mind and Brain Research, Department of Psychiatry and Psychotherapy CCM, Berlin Institute of Health, Humboldt University of Berlin, Corporate Member of the Free University of Berlin, Charité – Berlin University of Medicine, Berlin, Germany; ^2^QUEST – Center for Transforming Biomedical Research, Berlin Institute of Health, Berlin, Germany

**Keywords:** neurolaw, neuroscience evidence, responsibility, culpability, psychopathy

## Abstract

**Aim of the Study:**

The study examines how neurobiological and genetic explanations of psychopathy influence decision-making of German law students about legal and moral responsibility and sentencing of a defendant in a case of manslaughter. Previous studies from the United States and Germany have been criticized because they partly contradict legal analyses of real-world criminal cases. With a modified design, which integrates the main criticism, we re-examined the impact of biological explanations for psychopathy on decision-making in the courtroom.

**Methods:**

We developed an improved quasi-experimental design to probe three case vignettes presenting different explanations of psychopathy in a criminal case of manslaughter. All three vignettes present the same information about a forensic expert’s testimony that is said to report compelling evidence for the diagnosis of “psychopathy.” The independent variable being manipulated is the type of information supporting the expert diagnosis: either no biological explanation of “psychopathy” versus a neurological explanation (brain injury) versus a genetic explanation (MAOA gene). The outcome measure is a questionnaire on legal and moral responsibility, free will, the type of custody, and the duration of the sentence. The study is adequately powered. We openly publish the data and all statistical analyses as reproducible R scripts.

**Results:**

The answers of German law students (*n* = 317) indicate that the omission of a neurobiological explanation is significantly associated with higher ratings of legal responsibility while compared to no biological explanation. However, there was no significant difference on the prison sentencing and type of custody assigned. Furthermore, there was no difference in the self-reported impact of the explanation of psychopathy on the participants’ decision-making.

**Conclusion:**

Our findings from German law students corroborates previous research on German judges but is markedly distinct from studies on United States judges. Whereas in the United States, biological information seems to have a mitigating effect, it seems to increase the rate of involuntary commitment to forensic psychiatric hospitals in Germany.

## Introduction

Both United States courts and commentators have discussed the use of neuroscientific and genetic evidence in criminal cases as a “double-edged sword” for the defendant (Denno, 2012). On the one side, such evidence has a mitigating potential because it reduces the culpability of the defendant. On the other side, it can be an aggravating factor because it supports the assumption of future dangerousness. However, the United States legal theorist Denno (2015), who has analyzed hundreds real criminal cases, in which biological evidence was introduced, calls the double-edged sword theory a myth.

Indeed, neuroscience and genetic evidence is increasingly being introduced in criminal cases in the United States (Denno, 2012, 2015; Denno and McGivney, 2013; Farahany, 2015), in Canada (Chandler, 2015), Western Europe (Catley and Claydon, 2015; De Kogel and Westgeest, 2015), and Australia (Alimardani and Chin, 2018). In most of these cases, clinically established techniques such as EEG, structural brain imaging, and positron emission tomography have been used to demonstrate brain damage, whereas fMRI and neurogenetics have been used only in few cases (Fuss, 2016).

This paper contributes to the debate about the double-edged sword theory. First, we review the debate about the nature and the causes of psychopathy, and discuss its particular importance for criminal justice. Then we summarize the results of experimental studies investigating the double-edged sword effect.

The main part of the paper presents the results of our own experimental study that has investigated how neurobiological and genetic explanations of psychopathy influence the decision-making of German law students about legal and moral responsibility and sentencing of a defendant in a case of manslaughter. Our own study is based on older studies, but we have modified the design in order to integrate the main criticism of these studies.

Finally, we discuss the reasons for the inconsistent results of the different studies in the light of studies which have comprehensively analyzed real criminal cases in different countries. We suggest that the question whether neuroscientific and genetic evidence in criminal cases is a double-edged sword cannot be answered in general. Rather, the answer depends strongly on the system of criminal justice of a given country.

### The Nature and the Causes of Psychopathy and Its Particular Importance for Criminal Justice

Psychopathy is in the focus of neuroscientific and genetic research, although after a long and controversial debate (Crego and Widiger, 2015), it was not included as a stand-alone personality disorder in the DSM-5 (American Psychiatric Association, 2013).

The focus on psychopathy is justified because psychopathy is “one of the strongest dispositional predictors of aggression and violence” (Reidy et al., 2015). Psychopaths commit the most severe acts of violence; they commit twice as many violent crimes as non-psychopathic offenders and their risk of violent recidivism is at least five times higher (Reidy et al., 2015).

According to the influential psychopathy researcher Hare (1996, p. 25), psychopathy is “a devastating disorder defined by a constellation of affective, interpersonal and behavioral characteristics, including egocentricity; impulsivity; irresponsibility; shallow emotions; lack of empathy, guilt or remorse; pathological lying; manipulativeness; and the persistent violation of social norms and expectations.” Hare (1996, p. 26) describes psychopaths as “intraspecies predators who use charm, manipulation, intimidation, and violence to control others and to satisfy their own selfish needs. Lacking in conscience and in feelings for others, they cold-bloodedly take what they want and do as they please, violating social rules without the slightest sense of guilt or regret.”

For the diagnosis of psychopathy, forensic psychiatrists mostly use the Hare (2003) Psychopathy Checklist-Revised (PDL-R) and its derivatives (Reidy et al., 2015).

There are two opposing perspectives on psychopathy: (1) psychopathy is a mental disorder based on structural and functional dysfunctions of several brain areas, and (2) the developmental form of psychopathy is a moral or social disorder, but not a biological disorder.

Blair (2013) promotes the first perspective by describing psychopathy as a developmental disorder characterized by pronounced emotional deficits marked by reduction in guilt and empathy, and increased risk for displaying antisocial behavior. Blair (2013) emphasizes that psychopathy is not equivalent to antisocial personality disorder from the diagnostic systems DSM-IV-R or ICD-10, which focus on the antisocial behavior rather than underlying causes, i.e., the emotion dysfunction. Blair (2013) has suggested that the emotion dysfunction relates to three core functional impairments: the association of stimuli with reinforcement, the representation of expected value information and prediction error signaling. He hypothesizes that these functional impairments relate to the observed dysfunction seen in structural and functional MRI studies within the amygdala, vmPFC, and (only in youth populations) striatum (Blair, 2013).

Reimer (2008) suggests describing psychopathy without the language of disorder. According to an evolutionary model, psychopathy represents an alternative genetic strategy that is successful only at a particular low relative frequency in the population (Reimer, 2008). This idea is supported by game-theoretical models of non-cooperators who move between groups and “prey” on naïve cooperators (Dugatkin, 1992). This idea has been elaborated in sociobiology. Mealey (1995) explained sociopathy as “the expression of a frequency-dependent life strategy which is selected, in dynamic equilibrium, in response to certain varying environmental circumstances.” Reimer (2008) suggests that psychopaths are not disordered in any biological sense, but only different from the majority of people. Psychopaths are not impaired, but especially capable. They have a “pro-individual personality” with special capacities for “successful individualization” (Reimer, 2008). Particularly, they are capable of ignoring the distress of others and are better able to resist attempts at “moral” social reinforcing (Reimer, 2008). With regard to the amygdala-dysfunction theory of psychopathy, Reimer (2008) does not deny the role of the amygdala. Rather she says that the special development of the amygdala enables the “pro-individual personality” to successfully pursue the person’s goals, including reproductive ones, “without the hindrances imposed by other regarding norms” (Reimer, 2008). In this way, the “pro-individual personality” is able to insure the dissemination of her pro-individual genes in future generations (Reimer, 2008).

The view that psychopathy is a moral disorder that is not caused by a lack of capacities is supported by a study suggesting that psychopaths do understand the distinction between right and wrong, but do not care about such knowledge or the consequences that ensue from their morally inappropriate behavior (Cima et al., 2010).

Particularly the fact that many psychopaths are successful supports Reimer’s suggestion to describe psychopathy without the language of disorder. Babiak and Hare (2006) found a higher rate of psychopaths in the business world than in the general population (3.5% vs. 0.6–1%). Although both successful (not incarcerated) and unsuccessful (incarcerated) psychopaths show autonomic hyporeactivity (low resting heart rate), reduced emotional empathy, risky decision making and sensation-seeking, the successful psychopaths seem to have intact or even enhanced neurobiological functioning, which enables them to lie, con and manipulate successfully (Gao and Raine, 2010). In contrast, unsuccessful psychopaths have more cognitive and emotional deficits and tend to violent offending instead of white collar criminality (Gao and Raine, 2010).

In 1996, Hare (1996) noted that in most jurisdictions, psychopathy is considered an aggravating rather than a mitigating factor in determining criminal responsibility. However, research evidence explaining psychopathy in terms of an affective deficit, a thought disorder or brain dysfunction might lead some to view psychopathy as a mitigating factor (Hare, 1996). Hare (1996) considers a psychiatrist’s speculation that psychopathy would perhaps become “the kiss of life rather than the kiss of death” in first-degree murder cases, as “appalling, because psychopaths are calculating predators whose behavior must be judged by the rules of the society in which they live.”

The causes of psychopathy are controversial. Early studies investigated correlations between physiological indices such as heart rate and electrodermal activity with aggression, psychopathy/sociopathy, and conduct problems (Lorber, 2004). Low autonomic activity might contribute to the development of antisocial and criminal behavior, because it is a marker for fearlessness, and leads to sensation-seeking behavior (Raine, 2002).

Prenatal factors also contribute to antisocial and violent behavior, particularly pregnancy complications, birth complications, maternal smoking and alcohol consume during pregnancy; these factors strongly interact with each other (Raine, 2002).

Current research concentrates on the neurotransmitters serotonin, dopamine and vasopressin, the steroid hormones testosterone and cortisol, and brain structure and function (Rosell and Siever, 2015). Particularly, the amygdala, the prefrontal cortex and the striatum are in the focus of research (Rosell and Siever, 2015). However, the phenomenological heterogeneity of aggression is a source of inconsistencies between studies, and the categorical nature of psychiatric diagnoses is another critical issue (Rosell and Siever, 2015).

Sociopathy or chronic antisocial behavior can be a developmental or an acquired disorder (Mendez, 2009). The most famous case of acquired sociopathy caused by brain injury certainly is Phineas Gage. This case has become a scientific myth, perhaps because it is fascinating to watch someone break bad (Kean, 2014). Focal lesions affecting vmPFC and adjacent OFC/VLPFC include strokes, trauma, tumors, infections, and a ruptured anterior commissure aneurysm, and can lead to alterations in social and moral behavior (Mendez, 2009).

A meta-analysis of 43 structural and functional imaging studies showed significantly reduced prefrontal structure and function in antisocial individuals (Yang and Raine, 2009). A study with 56 males showed that men with lower amygdala volume exhibited higher levels of aggression and psychopathic features from childhood to adulthood (Pardini et al., 2014).

A systematic mapping of lesions with known temporal association to criminal behavior has revealed that the lesion sites are spatially heterogenous, including the medial prefrontal cortex and the orbitofrontal cortex. However, all these lesions are part of a unique functionally connected brain network, which is involved in moral decision making (Darby et al., 2018).

Evidence from behavioral genetics supports the conclusion that a significant amount of the variance in antisocial personality is due to genetic contributions. A meta-analytic review on behavioral genetic etiological studies of antisocial personality and behavior showed that 56% of the variance of antisocial personality and behavior can be explained through genetic influences, with 11% due to shared non-genetic influences and 31% due to unique non-genetic influences (Ferguson, 2010).

Particularly prominent is the MAOA gene, which is located on the X-chromosome. It encodes the MAOA enzyme, which metabolizes norepinephrine, serotonin and dopamine (Caspi et al., 2002). In males, a point mutation in the MAOA gene, which causes a complete MAOA deficiency, is associated with abnormal aggressive behavior and impulsivity in a large Dutch kindred (Brunner et al., 1993).

Caspi et al. (2002) found in males a gene × environment interaction between the MAOA gene and childhood maltreatment. Maltreated male children with high MAOA activity were significantly less likely to develop child conduct disorder, a disposition toward violence, an adult antisocial personality disorder and convictions for violent offenses (Caspi et al., 2002). Although the low-MAOA genotype on its own did not significantly increase the risk of developing antisocial behavior, it increased the risk for developing antisocial behavior among males who suffered maltreatment (Caspi et al., 2002).

Another research group replicated the results of Caspi’s study through the investigation of another sample of boys and a meta-analysis (Kim-Cohen et al., 2006).

A Finnish prisoner study with over 500 offenders revealed that a MAOA low-activity genotype and the CDH13 gene are associated with severe recidivistic violent behavior (Tiihonen et al., 2015).

However, a recent systematic meta-analysis did not find any significant association between any polymorphism analyzed, and aggression and violence; even subgroup analyses did not show any consistent findings (Vassos et al., 2014). Since no gene of major effect for aggression has been identified, the authors of the meta-analysis consider any approach to use genetic markers for risk prediction or to mitigate criminal responsibility questionable (Vassos et al., 2014). Tiihonen and coauthors emphasize, too, that the sensitivity and specificity of the genotype findings are much too low for any screening purposes for prevention of violent offending, and that putative risk factors such as genotype do not have a legal role in judgment about offenders (Tiihonen et al., 2015).

The relationship between genes and aggressive and antisocial behavior is much more complex than formerly believed. On the one hand, behavioral genetics shows that distinct polymorphisms of genes, which code for proteins controlling neurotransmitter function, are associated with individual vulnerability to aversive experiences, and may result in an increased risk of developing psychopathologies associated with violence (Palumbo et al., 2018). On the other hand, epigenetic studies indicate that aversive experiences particularly during prenatal life, infancy and early adolescence can introduce lasting epigenetic marks in genes, thus favoring the emergence of dysfunctional behaviors, including exaggerated aggression (Palumbo et al., 2018).

In the development of violent behavior and aggression, biological, psychodynamic and social factors play a role (Sopromazde and Tsiskaridze, 2018). Social and biological factors do not have simply an additive effect; rather the presence of both factors exponentially increases the rates of antisocial and violent behavior (Raine, 2002). In a good social environment, the association between biological factors and antisocial behavior is stronger (Raine, 2002).

Maltreatment during childhood and maternal withdrawal in infancy are significantly associated with antisocial personality disorder (Shi et al., 2012). The Cambridge Study in Delinquent Development, a prospective longitudinal study, which started in 1961, suggested that “the best predictors of psychopathy” were “having a convicted parent, physical neglect, low paternal involvement, low family income, and coming from a disrupted family” (Reidy et al., 2015). The transmission of psychopathy is mediated by psychosocial factors, namely the fathers’ employment and accommodation problems, and drug use (Auty et al., 2015).

### Experiments to Investigate the Double-Edged Sword Theory

For exploring the influence of neurobiological or genetic evidence on judging in criminal cases, several experimental studies with both mock jurors and judges have been performed. All but one of the studies described below are from the United States; only one study comes from Germany (Fuss et al., 2015).

Gurley and Marcus performed the first controlled study to examine the influence of neuroimages and neurological testimony on students’ verdicts in non-guilty by reason of insanity cases (Gurley and Marcus, 2008). They found that defendants diagnosed with psychosis were more likely to be judged non-guilty by reason of insanity than those diagnosed with psychopathy. Furthermore, the addition of neuroimages showing brain damage increased the likelihood of such a verdict, as did testimony stating that the defendant’s disorder began after a brain injury in a car accident (Gurley and Marcus, 2008).

Greene and Cahill (2012) performed a similar experiment with psychology students acting as mock jurors in a capital case. Consistent with the findings of Gurley and Marcus (2008), they found that both types of neuroscientific evidence had a mitigating effect by reducing the likelihood that jurors would sentence the defendant to death (but only for defendants at high risk of future dangerousness).

Appelbaum and Scurich (2014) investigated the influence of different explanations of impulsivity on the sentencing of jurors that were representative for the United States population. They found that evidence of genetic predisposition for impulsive behavior, including violence, did affect neither whether the defendant was convicted of first- or second-degree manslaughter or first- or second-degree murder, nor the sentence (Appelbaum and Scurich, 2014). However, participants who received evidence of childhood abuse or evidence of childhood abuse plus evidence of genetic predisposition imposed longer sentences (Appelbaum and Scurich, 2014). Genetic evidence and genetic plus childhood abuse evidence engendered the greatest fear of the defendant (Appelbaum and Scurich, 2014).

Recently, Allen et al. (2019) published a modified study design in order to distinguish between different motivations for punishment. They assumed that the question whether a given biological or psychological disorder is treatable has a high impact on juror’s decision for the type of custody and for the sentence duration (Allen et al., 2019). They found that both brain evidence and psychological evidence had mitigating effects on prison sentencing, whereby brain evidence had a stronger effect (Allen et al., 2019). However, brain evidence led to decisions for longer involuntary hospitalizations. They found that the variation in sentencing was explained best by “deontological considerations pertaining to moral culpability” (Allen et al., 2019).

Aspinwall et al. (2012) authors were the first to test experimentally the influence of genetic evidence on sentencing decisions of United States judges. In a nationwide experiment, they presented U.S. state trial judges (*N* = 181) a hypothetical case vignette, which was a modification of the famous case of *Mobley v. State* (Mobley v. The State, 1995; Mobley v. Head, 2001). In the case vignette, the offender was convicted of aggravated battery (instead of murder as in the real case). All participants received a psychiatric testimony about the offender’s psychopathy. The study used a 2 × 2 design. One group was told that the psychiatric testimony was presented by the defense; the other one that it was presented by the prosecution. One group received the explanation that the offender’s psychopathy was related to his low-activity MAOA genotype; the other group did not receive any genetic explanation. The judges were randomly assigned to one of these four groups. The authors found that the judges considered the psychiatric testimony about psychopathy aggravating. The additional presentation of neurogenetic evidence for the offender’s psychopathy significantly reduced sentencing (from 13.9 to 12.8 years) (Aspinwall et al., 2012).

Fuss et al. (2015) repeated Aspinwall’s study in order to investigate whether the double-edged sword effect can also be found in German judges. They found that neurogenetic evidence significantly reduced the German judges’ estimation of legal responsibility of the convict. Nevertheless, the average prison sentence was not influenced. Most interestingly, neurogenetic evidence presented by the prosecution significantly increased the number of judges (23% compared with 6%) ordering an involuntary commitment in a forensic psychiatric hospital (Fuss et al., 2015). The different results of these two studies show that the judges’ responses to neurogenetic evidence is highly influenced by the legal system in which they operate (Fuss et al., 2015).

The legal theorists Denno and McGivney (2013) have strongly criticized Aspinwall’s study as significantly flawed due to problems with both the design and the methodology. Their main points of criticism are: (1) The hypothetical defendant is featured with psychopathy, although this condition is not fully recognized in the medical community and not listed in the current or any prior edition of the DSM. Indeed, the defendant in the real-life case upon which the study’s hypothetical case is based claimed that he had an antisocial personality disorder. (2) The study authors instructed the participants that rehabilitation was not an alternative for the offender, because treatment has been ineffective for adult psychopaths so far. This directive substantially loaded the dice in favor of the judges’ sentencing decisions being influenced by considerations of future dangerousness or retribution. (3) The study did not include a control group, which was not told that the offender was diagnosed with psychopathy. (4) In contrast to the real-life case, the study’s defendant did not commit murder, but only an aggravated assault. Insofar, the study’s hypothetical case differs significantly from a typical behavioral genetics criminal case, which involve capital crimes. (5) The study does not describe the gene-environment interaction that is present in nearly any real-world criminal case involving behavioral genetics evidence (Denno and McGivney, 2013).

Denno and McGivney (2013) conclude that Aspinwall’s study may interpret the effects of genetics evidence as a double-edged sword, but that there is no support for such a simplistic perspective in actual case law nor are the evidentiary hurdles the same for each side of that sword. It is much more difficult for the State to prove that genetic factors will predict a defendant’s future dangerousness than it is for the defense to introduce such information to suggest why a defendant should not be executed (Denno and McGivney, 2013).

Denno and McGivney (2013) emphasize that Denno’s (2012) comprehensive survey of criminal cases involving behavioral genetics evidence did not reveal a single case in which such evidence was used to support the likelihood of a defendant’s future dangerousness. According to Denno’s survey, there was no case in which the State introduced behavioral genetics evidence in any capacity, much less as an aggravating factor. To the contrary, only defense attorneys introduced behavioral genetics evidence into court (Denno and McGivney, 2013).

### Objective and Conception of the Present Study

The main objective of this study is to investigate the influence of different types of neurobiological explanations on the sentencing decisions made by German law students. In particular, we wanted to find out whether and to what extent neurobiological explanations influence the students when it comes to evaluating the legal and moral responsibility of a psychopathic offender, deciding about a prison sentence or forensic psychiatric hospital confinement, and to sentencing. Thereby, we compared two different neurobiological explanations (namely, a genetic explanation and a brain injury explanation) with no neurobiological explanation.

The present study is based on the studies of Aspinwall (Aspinwall et al., 2012) and Fuss (Fuss et al., 2015). However, we modified their concept in order to address some of Denno and McGivney’s (2013) criticism.

First, we presented a case of manslaughter (as in the real case *Mobley v. State*) instead of aggravated assault (as in the studies of Aspinwall and Fuss), because most real criminal cases, in which genetic evidence is presented, are capital crime cases.

Second, we did not establish two groups of which one was told that the genetic evidence was presented as mitigating by the defense, and the other one was told that it was presented as aggravating by the prosecution. The latter case is unrealistic according to Denno’s surveys (Denno, 2012, 2015). Particularly, for Germany, this case is unrealistic.

Third, we established three different groups: the first group received genetic evidence, the second group received neurobiological evidence for a brain trauma, and the third group did not receive any biological evidence.

A further difference is that we interviewed law students instead of judges. The main reason for this decision was that we wanted to achieve a high response rate. We estimate that the response rate among German judges in the study of Fuss (Fuss et al., 2015) is only about 2%. [Only 375 judges responded, although in 2016, there were more than 15,000 judges at ordinary courts in Germany (Statista, 2018).] Due to the extreme lack of judges and the severe overload of the German courts, we expected that even fewer judges would participate in a new survey among judges. A small response rate is generally associated with a strong bias. In order to receive a high response rate, we decided for investigating law students instead of judges. In our experience, nearly all students participate in surveys, which are recommended by their professors and conducted directly after the courses. A further reason for investigating law students was that they are the future decision-makers in criminal cases, and they are particularly influenced by university professors and thus by recent developments in legal theory.

## Materials and Methods

### Participants

We recruited 317 law students from three major German universities in the summer semester of 2018. We invited the students after the lecture classes to participate in the survey. They did receive neither course credit nor an allowance for participating. We informed the students about the voluntariness of participation, about the study purposes and procedures, which guaranteed full anonymity and compliance with the EU General Data Protection Regulation. Ethics approval by the Local Ethics committee of Charité – Universitätsmedizin Berlin was not applicable given the study design, purpose, and procedures.

### Design

This prospective quasi-experimental study used case vignettes as independent variables (Aguinis and Bradley, 2014; Auspurg and Hinz, 2014). We developed a specific case vignette to assess the influence of three different explanations of psychopathy in a criminal case of manslaughter. All three vignettes contain the same information about a forensic expert’s testimony that is said to report compelling evidence for the diagnosis of “psychopathy.” The independent variable being manipulated is the type of information supporting the expert diagnosis: either no biological explanation of “psychopathy” versus a neurobiological explanation (brain injury) versus a genetic explanation (MAOA gene). All three vignettes contained the same set of instructions and background information based on the German Penal Code. The outcome measure is a questionnaire on legal and moral responsibility, free will, the type of custody, and the duration of the sentence. The vignettes and the questionnaire can be found in the Supplementary Material.

**FIGURE 1:**
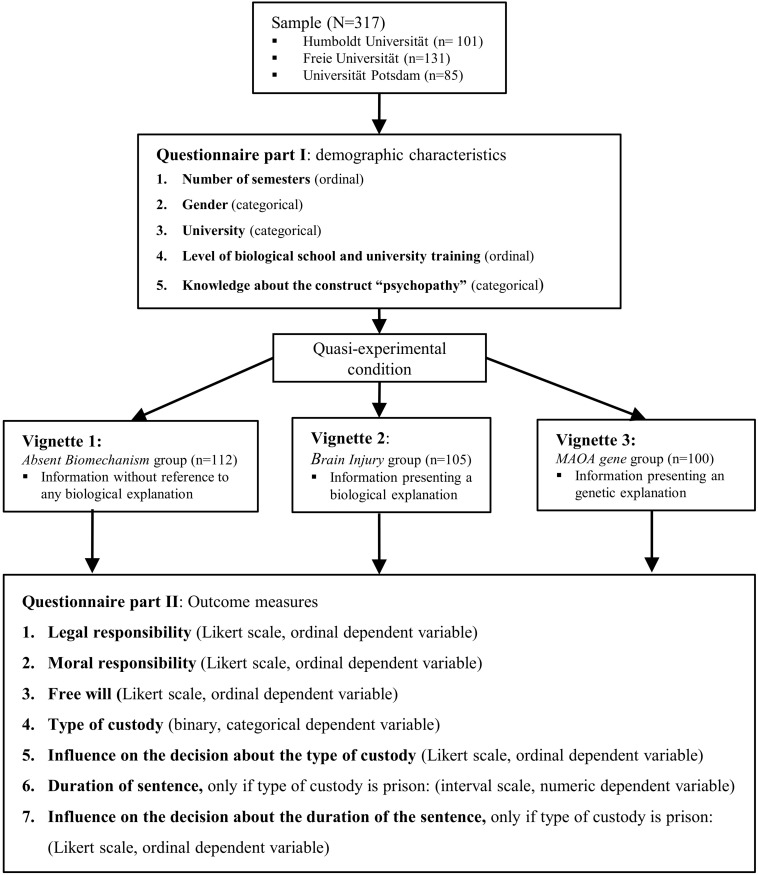
Flow chart displaying the experimental design of this vignette study and the participant flow as required by the CONSORT statement (Schulz et al., 2010).

Participants from all three universities were allocated to three experimental conditions (Figure 1). Each participant received a questionnaire asking for demographic information and presenting one of three types of vignettes. Each vignette initially presented the exact same content and phrasing of a criminal case. The case describes a young man who committed manslaughter of his former girlfriend. All vignettes reported that a psychiatric expert had assessed the perpetrator as a psychopath. Each vignette gave a different etiological explanation for the psychopathy of the perpetrator, depending on the experimental condition. The full text of the vignettes is presented in the Supplementary Material. Participants received all textual information in German translated by a German native speaker (S.M.). We collected the data in the form of a paper and pencil questionnaire.

For analytical reproducibility, we openly publish the statistical analyses as R scripts together with the full data set on the Open Science Framework website^1^.

### Statistical Analysis

We conducted seven separate fixed-effect Analyses of Variance (ANOVA) to examine group differences (main effect). The seven dependent variables are (1) legal responsibility, (2) moral responsibility, (3) free will, (4) the type of custody assigned, (5) the duration of sentencing, (6) the influence of the biological explanation on the type of custody assigned, and (7) the influence of the biological explanation on the duration of sentencing.

As between-subjects factor we used group allocation (“Absent Biomechanism” group versus “Brain Injury” group versus “MAOA Gene” group). We included all demographic variables consisting of gender, number of semesters, level of biology training, the acquaintance with psychopathy and home university as between-subject factors into the analyses. For main effects of group differences, a strict alpha-level of 0.005 was used due to multiple testing of a family of related hypotheses about the influence of the case vignette on the judgment of the law students and the associated risk of an inflated false-positive rate (Benjamin et al., 2018).

*Post hoc t*-tests were performed in the event of significant group differences according to the conventional alpha-level of 0.05 but were considered “exploratory” if above the predefined alpha-level for the main effects (0.005). With “exploratory,” we mean that the effect warrants replication but can be considered suggestive to devise new hypotheses. The multiple *post hoc* pairwise-comparisons can determine between which specific pairs of groups, the difference of the means is statistically significant. Given unequal group sizes, the non-parametric Games–Howell test was chosen over the more common Tukey’s *post hoc* test because the former does not make assumptions about normality, equal variances, or sample sizes.

For ANOVA, partial η^2^ was used as effect size (small effect ≥ 0.01; medium effect ≥ 0.06; large effect ≥ 0.14). Exploratory χ^2^-tests were used to examine potential differences in demographic characteristics between the three groups (Table 1).

**TABLE 1:** Comparison of baseline characteristics between the groups.

	**Group**					
				
	**Absent**	**Brain injury**	**MAOA gene**	**Row sum**	
**Gender**					
Female	57(18.4%)	55(17.8%)	60(19.4%)	172(55.6%)	χ*^2^*(2, *N* = 309) = 2.082, *Cramer’s V* = 0.082, *p* = 0.353
Male	54(17.5%)	45(14.6%)	38(12.3%)	137(44.4%)	
Total	111(35.9%)	100(32.4%)	98(31.7%)	309(100%)	
**University**					
Humboldt-Universität zu Berlin	34(10.7%)	44(13.9%)	23(7.3%)	101(31.9%)	χ^2^(4, *N* = 317) = 14.76, *Cramer’s V* = 0.153, *p* = 0.005
Freie Universität Berlin	55(17.4%)	30(9.5%)	46(14.5%)	131(41.4%)	
Universität Potsdam	23(7.3%)	31(9.8%)	31(9.8%)	85(26.9%)	
Total	112(35.3%)	105(33.1%)	100(31.5%)	317(100%)	
**Level of Biology training**					
Grammar school until 10th grade	9(2.8%)	14(4.4%)	12(3.8%)	35(11%)	χ*^2^* (4, *N* = 317) = *19.272, Cramer’s V* = 0.174, *p* = 0.001
Until university entrance diploma	83(26.2%)	89(28.1%)	82(25.9%)	254(80.2%)	
University classes (Biology/Medicine)	20(6.3%)	2(0.6%)	6(1.9%)	28(8.8%)	
Total	112(35.3%)	105(33.1%)	100(31.5%)	317(100%)	
**Semester**					
First	2(0.6%)	3(0.9%)	0(0%)	5(1.5%)	χ*^2^*(18, *N* = 317) = 32.283, *Cramer’s V* = 0.226,
Second	53(16.7%)	28(8.8%)	46(14.5%)	127(40%)	*Fisher’s p* = 0.005
Third	0(0%)	2(0.6%)	2(0.6%)	4(1.2%)	
Forth	56(17.7%)	64(20.2%)	46(14.5%)	166(52.4%)	
Fifth	0(0%)	2(0.6%)	0(0%)	2(0.6%)	
Sixth	1(0.3%)	3(0.9%)	3(0.9%)	7(2.1%)	
Seventh	0(0%)	1(0.3%)	0(0%)	1(0.3%)	
Eighth	0(0%)	0(0%)	2(0.6%)	2(0.6%)	
Eleventh	0(0%)	0(0%)	1(0.3%)	1(0.3%)	
Twelfth	0(0%)	2(0.6%)	0(0%)	2(0.6%)	
Total	112(35.3%)	105(33.1%)	100(31.5%)	317(100%)	
**Acquaintance with psychopathy**					
Nothing at all	28(9%)	17(5.5%)	16(5.1%)	61(19.6%)	χ*^2^*(12, *N* = 311) = 20.706, *Cramer’s V* = 0.182,
Movies	3(1%)	8(2.6%)	3(1%)	14(4.6%)	*Fisher’s p* = 0.051
Fictional literature	5(1.6%)	3(1%)	2(0.6%)	10(3.2%)	
School	8(2.6%)	6(1.9%)	5(1.6%)	19(6.1%)	
Popular science magazines	6(1.9%)	5(1.6%)	1(0.3%)	12(3.8%)	
TV documentations	28(9%)	28(9%)	17(5.5%)	73(23.5%)	
Scientific literature	34(10.9%)	36(11.6%)	52(16.7%)	122(39.2%)	
Total	112(36%)	103(33.1%)	96(30.9%)	311(100%)	

Missing values arising from incomplete survey responses were less than 3% and imputed using non-parametric random forests (Stekhoven and Bühlmann, 2012). *A priori* Power to detect a medium effect size or larger in a balanced three group ANOVA with α = 0.005 and a Power = 90% was estimated to be optimal with a total sample size of *n* = 318.

All statistical analyses were carried out in R version 3.5.2.

## Results

In total, 317 law students returned the questionnaire at least partially answered. Overall, 1.2% of the questionnaire response are incomplete. Most participating law students were enrolled at the Freie Universität Berlin (41.4%), followed by Humboldt-Universität zu Berlin (31.9%) and Universität Potsdam (26.9%). 55.6% of the law students were female and 44.4% were male. Table 1 shows the distribution of the sample characteristics between the experimental groups.

### Moral Responsibility

**FIGURE 2:**
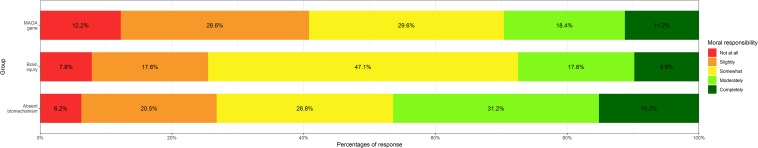
Plot of proportional cross-tables (contingency tables) showing the response to the question how morally responsible the offender is.

*Fixed-Effects* ANOVA indicate no significant differences between the groups according to our predefined criterion of significance [*F*(2, 294) = 5.15, *p* < 0.006, $\eta_{p}^{2}$ = 0.03]. The partial η^2^ = 0.03 and 90% CI suggest that this effect is of small effect size [0.01, 0.07]. Exploratory *post hoc t*-tests revealed no significant differences between the “Absent Biomechanism” group and the “Brain Injury” group [*t*(214.79) = 1.80, *p* = 0.171], nor between the “Brain Injury” group and the “MAOA gene” group [*t*(196.16) = 1.08, *p* = 0.527], or the “MAOA gene” group and the “Absent Biomechanism” group [*t*(204.98) = 2.71, *p* = 0.020] (Figure 2).

### Free Will

**FIGURE 3:**
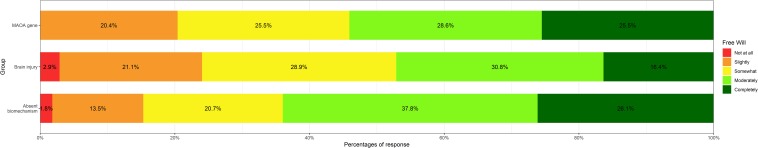
Plot of proportional cross-tables (contingency tables) showing the response to the question to which degree the offender had a free will at the time of manslaughter.

*Fixed-Effects* ANOVA indicate no significant differences between the groups [*F*(2, 294) = 3.95, *p* < 0.02, $\eta_{p}^{2}$ = 0.03]. The partial η^2^ = 0.03 and 90% CI suggest that this effect is of small effect size [0.00, 0.06]. Exploratory *post hoc t*-tests revealed no significant differences between the “Absent Biomechanism” group and the “Brain Injury” group [*t*(212.85) = 2.42, *p* = 0.043], nor between the “Brain Injury” group and the “MAOA gene” group [*t*(204.94) = 1.17, *p* = 0.475], or the “MAOA gene” group and the “Absent Biomechanism” group [*t*(202.35) = 1.17, *p* = 0.470] (Figure 3).

### Legal Responsibility

**FIGURE 4:**
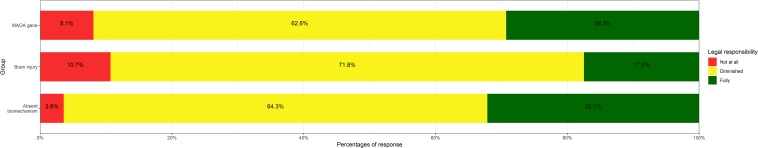
Plot of proportional cross-tables (contingency tables) showing the response to the question how legally responsible the offender is.

*Fixed-Effects* ANOVA indicate significant differences between the groups [*F*(2, 294) = 8.24, *p* < 0.001, $\eta_{p}^{2}$ = 0.05]. The partial η^2^ = 0.05 and 90% CI suggest that this effect is of small to possibly moderate effect size [0.02, 0.10]. *Post hoc t*-tests revealed significant differences between the “Absent Biomechanism” group and the “Brain Injury” group [*t*(213.04) = 3.27, *p* = 0.004], i.e., the group that received no biological explanation assigned a higher legal responsibility. The mean response in the “Absent Biomechanism” group is 2.29 (SD = 0.53) and in the “Brain Injury” group 2.05 (SD = 0.54) with the response “1” meaning “not at all legally responsible,” “2” being “diminished legally responsible,” and “3” being “fully legally responsible.” Thus, most students answered “diminished legally responsible,” but in the “Brain Injury” group significantly more students considered the perpetrator “not at all” legally responsible compared to the “Absent Biomechanism” group. In the latter group, significantly more students responded “fully legally responsible” compared to the “Brain Injury” group (Figure 4).

*Post hoc t*-tests revealed no significant differences between the “MAOA gene” and the “Brain Injury” group [*t*(200.53) = 2.20, *p* = 0.074], nor between the “Absent Biomechanism” group and the “MAOA gene” group [*t*(201.43) = 0.86, *p* = 0.666].

### Type of Custody

**FIGURE 5:**
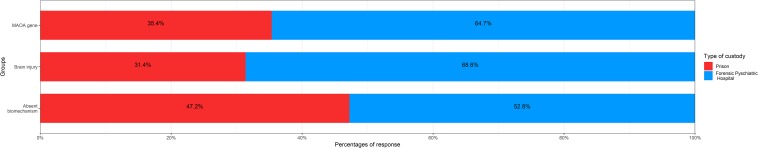
Plot of proportional cross-tables (contingency tables) showing the response to the question, which type of custody should be assigned to the offender depending on the group of respondents.

*Fixed-Effects* ANOVA indicate no significant differences between the groups [*F*(2, 302) = 4.12, *p* < 0.017, $\eta_{p}^{2}$ = 0.03]. The partial η^2^ = 0.03 and 90% CI suggest that this effect is of small effect size [0.00, 0.06]. *Post hoc t*-tests revealed no significant differences between the “Absent Biomechanism” group and the “Brain Injury” group [*t*(215.00) = 2.26, *p* = 0.063], nor between the “Brain Injury” group and the “MAOA gene” group [*t*(209.01) = 1.83, *p* = 0.163], or the “MAOA gene” group and the “Absent Biomechanism” group [*t*(202.05) = 0.39, *p* = 0.918] (Figure 5).

### Duration of Sentencing

On a descriptive level, the mean prison sentence assigned by law students differed only slightly and the group differences were not significant. In the “Absent Biomechanism” group, the mean prison sentence assigned was 9.15 years (SD = 3.47 years), in the “Brain Injury” group 10.06 years (SD = 5.37 years) and in the “MAOA Gene” group 10.54 years (SD = 3.84 years) (Table 2).

**TABLE 2:** Duration of sentencing depending on the group of respondents (M = mean, SD = standard deviation, *n* = number of students in the group).

**Group**	**M (SD) [years in prison]**	***n***
Absent biomechanism	9.15 (3.47)	53
Brain injury	10.06 (5.37)	33
MAOA gene	10.54 (3.84)	35

*Fixed-Effects* ANOVA indicate no significant differences between the groups [*F*(2, 106) = 0.64, *p* < 0.530, $\eta_{p}^{2}$ = 0.01]. The partial η^2^ = 0.01 and 90% CI suggest that this effect is of zero to very small effect size [0.00, 0.05].

For detailed descriptions of the results for all statistical analyses calculated, see Supplementary Statistical Analysis.

One plausible suggestion is that the level of expertise and background knowledge can influence decision-making. For this reason, we included variables such as the number of semesters of the participants, their home university, their level of biological training and their acquaintance with psychopathy as covariates in the fixed effect ANOVA. Doing so allowed us to study the influence of these factors on the outcome such as sentencing. However, in our sample, these factors did not had a significant impact [number of semesters: *F*(1, 106) = 0.06, *p* < 0.814; level of biological training: *F*(2, 106) = 0.03, *p* < 0.970; acquaintance with psychopathy: *F*(6, 106) = 0.37, *p* < 0.895]. We had a very homogeneous sample with 92.4% of the students being in semester 2 or 4, and with 80.2% having their biology knowledge from school until the university entrance diploma (Table 1). We only found an association between the level of biological training and the evaluation of moral responsibility, which we consider exploratory as the *p*-value is greater than *p* = 0.005 [*F*(2, 294) = 4.11, *p* < 0.017]. This may give rise to the hypothesis that the level of biological training affects the decision-making of law students with regard to the assessment of moral responsibility, but further research directly addressing this hypothesis would be needed to investigate this hypothesis.

### Influence of Expert Testimony on Decision-Making

We also examined whether participants noticed being influenced in their decision-making by the expert testimony. As described in further detail in the Supplementary Materials S6, S7, there was no significant difference between the participants’ responses of the three groups to the question whether the expert testimony affected the decision to assign a prison sentence or custody in a forensic hospital [*F*(2,302) = 0.38, *p* < 0.685.]. There was also no group difference with regard to the participants’ responses to the question whether the expert testimony affected the duration of prison sentencing assigned [*F*(2,105) = 0.69, *p* < 0.505].

## Discussion

Neuroscientific evidence has been increasingly introduced in criminal trials all around the world to explain criminal behavior. Our results indicate that the different neurobiological information has only small effects on the assessment of law students. However, neurobiological information is often used when very high stakes are involved such as death penalty or the verdict “not guilty” in capital crimes. In such contexts, every bit of information that influences human judgment plays a decisive role. In our study, the strongest effect was observed with regard to legal responsibility. Law students were asked to rate the legal responsibility of a perpetrator after having received one of three different kinds of information about the perpetrator. Overall, there was a significant difference in the assessment of legal responsibility of the law students depending on the kind of information received. Pair-wise comparisons of the groups showed that students who received information describing a major brain injury of the perpetrator rated the legal responsibility significantly lower than the students who did not receive a biological explanation. However, no similar effect was found for information describing a MAOA gene susceptibility for psychopathy.

Our results can be compared to previous findings of the studies of Aspinwall (Aspinwall et al., 2012) and Fuss (Fuss et al., 2015), although we modified the study design in light of Denno and McGivney’s (2013) criticism. Our sample size (*N* = 317) is comparable to the above-mentioned studies (Aspinwall et al., 2012: *N* = 181; Fuss et al., 2015: *N* = 372).

Similar to our main finding of reduced legal responsibility in case of a brain injury, the legal responsibility in the study of Fuss (Fuss et al., 2015) was significantly lower in the group that received biomechanistic information compared to no biological information.

In addition to legal responsibility, we examined the influence of neuroscientific evidence on law students’ assessment of moral responsibility, of free will, the type of custody, and of the duration of sentence. Due to the multiplicity of statistical analyses, we used a strict criterion for significance of *p* < 0.005 as recommended by Benjamin et al. (2018) and considered the *p* < 0.05 as exploratory. The effect of brain injury evidence on legal responsibility was the only effect that was significant given the strict criterion. However, on a more exploratory interpretation, we observed some suggestive differences of the influence of neuroscientific evidence on the students’ assessment of moral responsibility and free will. The law students who received biological information about the MAOA gene, tended to assign less moral responsibility compared to the students who received no biological explanation. In addition, the free will of the perpetrator was assessed to be lower by the group of students who received information about a brain injury compared to students who received no biological explanation. Due to the exploratory character of these analyses, these findings warrant replication in an independent sample before taken to represent real effects.

The mean prison sentence assigned by law students differed only slightly, and the group differences were not significant (“Absent Biomechanism” group: 9.15 years, “Brain Injury” group: 10.06 years, “MAOA Gene”: 10.54 years).

In comparison, in Aspinwall’s study (Aspinwall et al., 2012) the mean prison sentence was higher than in our sample. Important to note is that the mean prison sentence was lower in the group with genetic evidence (12.83 years) than in the group without biological explanation (13.93 years) (Aspinwall et al., 2012). In Fuss’ study (Fuss et al., 2015), the average prison sentence was not affected by the presentation of neurogenetic evidence. Also in our study, the prison sentence was not influenced by the presentation of biological evidence.

## Limitations and Strengths

The ecological validity of this study can be doubted, as well as that of all previous studies. As Scurich (2018) has recently pointed out, it is doubtful that a cursory written expert’s report is remotely similar to a real expert’s testimony who presents his results in court, uses PowerPoint presentations and is subjected to cross examination. For future studies, more realistic simulations should be used to increase the ecological validity.

This is a prospective study with experimental control to increase internal validity. Since law students are the upcoming judicial decision-makers in the legal system, the study is also externally valid in respect of the population examined. However, quasi-experimental study designs come with certain risk of bias. In particular, the lack of randomization prevents any strong claims ruling out that non-measured variables confound the results. For mitigating the risk of bias, we included theoretically relevant demographic differences between groups as between-factors into the statistical analysis. *Post hoc* tests were adjusted for multiple comparison and the statistical power was sufficient to find at least medium effects.

A strength of our paper is that we rigorously corrected the significance level for multiple testing (Benjamin et al., 2018).

A further strength is that our statistical analysis accounts for the influence of demographic factors such as gender, the number of semesters, and level of biological education.

## Conclusion

The main purpose of this research was to understand the judgments of German law students depending on two different types of neuroscientific evidence being presented in the courtroom. The question is whether biological information influences the judgment of law students. Indeed, the “Brain Injury” group evaluated the perpetrator less legally responsible than the “Absent Biomechanism” group.

The question whether neuroscientific and genetic evidence in criminal cases is a double-edged sword or not, has been answered differently by different studies. The results from the experimental studies from Germany (Fuss et al., 2015, and the present study) are partly inconsistent with the results from the experimental studies from the United States (Gurley and Marcus, 2008; Aspinwall et al., 2012; Greene and Cahill, 2012; Appelbaum and Scurich, 2014; Allen et al., 2019). In contrast to the United States studies, the German studies did not find a mitigating effect of neuroscientific evidence in terms of the duration of sentencing. The study of Fuss found that neurogenetics evidence leads to more decisions for forensic psychiatric hospital, which has the consequence of a longer and indefinite detention (Fuss et al., 2015).

For investigating whether a double-edged sword exists, it is important to compare the results of surveys of real criminal cases from different countries, too. Indeed, they provide mixed results, too.

For the United States, Denno has comprehensively analyzed criminal cases from the United States, of which 553 addressed neuroscience evidence for the defendant (from 1992 to 2002) (Denno, 2012), and 81 addressed behavioral genetics evidence (including family history evidence and MAOA deficiency evidence) (from 1994 to 2007) (Denno, 2015). Denno’s studies systematically investigated how United States courts assess the mitigating and aggravating effects of neuroscience or genetic evidence, respectively. She found that neuroscience evidence is typically raised in cases where defendants are facing the death penalty, a life sentence or a substantial prison sentence (Denno, 2015). Usually neuroscience evidence is offered to mitigate punishments in the way that traditional criminal law has always allowed, especially in the penalty phase of death penalty trials (Denno, 2015). Neuroscience evidence is only rarely used to bolster a defendant’s future dangerousness (Denno, 2015). In the rare cases when prosecutors utilized neuroscientific evidence to implicate a defendant’s propensity to commit crimes, they typically did so only by building upon the evidence first introduced by a defense expert (Denno, 2015). The same is valid for behavioral genetics evidence, which has been applied almost exclusively as mitigating evidence in death penalty cases (Denno, 2012). Between 2007 and 2011, the State never presented behavioral genetics evidence as aggravating evidence or for indicating the future dangerousness of the defendant (Denno, 2012). United States courts accept both neuroscience and behavioral genetics evidence (Denno, 2012, 2015). They even expect attorneys to raise neuroscience evidence when possible on behalf of their clients. Courts grant defendants their “ineffective assistance of counsel” claims when attorneys fail to pursue mitigating neuroscience or genetic evidence (Denno, 2015). Sometimes courts even penalize attorneys who neglect the obligation to pursue mitigating neuroscience evidence (Denno, 2015). Denno concludes that her study “controverts the popular image of neuroscience evidence as a double-edged sword – one that will either get defendants off the hook altogether or unfairly brand them as posing a future danger to society” (Denno, 2015).

Farahany (2015) also examined the use of neurological and behavioral genetic evidence in United States criminal law. For that, she and her team investigated 1,585 judicial opinions issued between 2005 and 2012. Although many scientists discredit the use of neurobiological evidence in criminal law, and some call for “an outright ban on its use” due to significant methodological problems, Farahany (2015) concludes that neuroscience is “already entrenched in the United States legal system.” She found that neurobiological evidence is increasingly used in criminal cases (Farahany, 2015). Neurobiological evidence is used broadly, and is not limited to capital cases as mitigating evidence (Farahany, 2015). Farahany states that neurobiological evidence is “in a rarified position of must-investigate evidence,” and summarizes: “Defense counsels are ineffective if they fail to mount a defense at all, sleep through an entire (but not just parts of) a trial, or if they fail to investigate a probable neurobiological abnormality in a defendant.” (Farahany, 2015).

In the Netherlands, neuroscientific and genetic evidence is in most cases no double-edged sword. According to De Kogel and Westgeest’s (2015) analysis of 231 criminal cases published between 2000 and 2012, neuroscientific evidence is introduced as mitigating evidence in the majority of the cases found. Only in some cases, defendants were considered diminished or not responsible for their crime, but received a longer sentence, such as a custody in a forensic psychiatric hospital that can be periodically extended. In some other cases, the defendants did not receive longer sentences despite their “untreatable” neurobiological deficits, when the experts saw room for reduction of recidivism risk (De Kogel and Westgeest, 2015).

For England and Wales, Catley and Claydon (2015) analyzed 204 criminal cases from 2005 to 2012. They found that most appellants, who used neuroscientific evidence when they appealed against conviction, were unsuccessful. However, in the few successful cases, the neuroscientific evidence had nearly always a central role in the successful appeal (Catley and Claydon, 2015). The authors do not discuss whether they found the double-edged sword effect in the cases analyzed.

However, in Canada, neuroscientific evidence is a double-edged sword for criminal offenders according to Chandler’s (2015) analysis of 133 criminal cases published between 2008 and 2012. In Canada, the most common form of biological evidence considered is fetal alcohol spectrum disorder, followed by medical history of traumatic brain injury and neuropsychological testing (Chandler, 2015). Functional MRI investigations and genetic tests did not play any role in the court decisions analyzed (Chandler, 2015). Chandler (2015) found that neuroscientific evidence suggested diminished capacity, but also tends to increase judgements about risk and dangerousness given the view that brain injuries can sometimes be managed but not cured.

For Australia, Alimardani and Chin (2018) found on grounds of a systematic review that in some cases, neuroscientific evidence presents a double-edged sword. It can serve to either aggravate or mitigate a sentence. Because the courts also consider the protection of society, a sentence can be prolonged when neuroscientific evidence suggests that the offender poses a particular risk of re-offending. On the other side, neuroscientific evidence can suggest a reduced risk of future offending, and thus support a more lenient sentence. Furthermore, neuroscientific evidence can mitigate the offender’s moral culpability and thus reduce the significance of general deterrence, so that the sentence can be mitigated. In most cases analyzed by the authors, neuroscience evidence only leads to mitigation and was rarely used as evidence for the offender’s risk of recidivism (Alimardani and Chin, 2018).

For Iran, Alimardani (2018) has investigated the potential applicability of neuroscientific evidence in the criminal justice system. He demonstrates that neuroscientific evidence can be used *inter alia* both for establishing the insanity defense and for mitigating the sentence for some kinds of crimes. He concludes that neuroscientific evidence can result on the one hand in a successful defense of insanity and thus in the offender’s discharge. On the other hand, if it indicates a condition, which can put the society in danger, the offender will be detained in a psychotherapeutic facility for an indeterminate period (Alimardani, 2018). Therefore, neuroscientific evidence may be a double-edged sword in the Iranian criminal justice system.

For Germany, an investigation of the influence of neuroscientific and genetic evidence in real criminal court cases has not been published so far to the best of our knowledge.

In summary, it can be said that neuroscience evidence can present a double-edged sword in Canada, Netherlands, Australia, and Iran, but not in the United States. However, even in the countries, in which a double-edged sword effect might occur, neuroscience evidence seems to lead more often to mitigation than to aggravation of sentencing.

Therefore, the question whether neuroscientific and genetic evidence in criminal cases is a double-edged sword, cannot be answered in general. Rather, the answer depends strongly on the given system of criminal justice. In the United States, punishment is much harder than in most other Western countries; particularly, other Western countries have abolished the capital punishment since many years. On the other side, in the United States, the standards for admitting mitigating evidence at sentencing are purposefully lax (Segal, 2016). The laxity in the admission of mitigating evidence could be the other side of the coin of extreme severity in sentencing.

Furthermore, the type of criminal justice system (common law system vs. civil law system) presumably has a significant impact on the influence of biological evidence on sentencing. Because the roles of professional and lay judges differ in the different systems, and because neuroscientific evidence influences the two groups of judges presumably in different ways.

In the common law system, the jury of lay judges decides whether to convict or to acquit the defendant, whereas the professional judge decides about the penalty (whether detention should take place in prison or in a forensic hospital and the length of the sentence). In the United States, in criminal law cases, in which the death penalty is a prospective sentence, a “death-qualified jury” has to be established. Such a jury has to be composed of jurors who will fairly consider all punishment options, including the death penalty and life imprisonment.

In the civil law system, a jury is called only in court cases involving serious criminal offenses. The jury’s influence on sentencing is much smaller than in the common law system. In Germany, in the case of homicide, the jury court consists of three professional and two lay judges. The lay judges are equal judges, who have a full say in the decision-making process about the guilt of the accused and subsequently on the sentence.

Therefore, it is necessary to carefully distinguish the results of studies, which have investigated the effect of biological evidence on the sentencing of professional judges vs. the sentencing of potential lay judges.

The improvement of the ecological validity of experimental research in this field should be in the focus of future research. For that, it is important that the experimental research learns from the research on real criminal cases and vice versa.

## Data Availability Statement

All datasets generated for this study are included in the manuscript/Supplementary Files.

## Ethics Statement

Ethical review and approval was not required for the study on human participants in accordance with the local legislation and institutional requirements. The participants provided their written informed consent to participate in this study.

## Author Contributions

DG: data collection. SM: study design. DG and MB: statistical analysis. SM, MB, and DG: writing of the report. SE: scientific advice. SM: revision of the manuscript and response to the reviewers.

## Conflict of Interest

The authors declare that the research was conducted in the absence of any commercial or financial relationships that could be construed as a potential conflict of interest.

## Footnotes

1 https://osf.io/6r5ng/

## Abbreviations

α: level of significance of the probability value
χ^2^: chi-squared
$\eta_{p}^{2}$: partial eta-squared
df: degrees of freedom
M: mean
MAOA: monoamine oxidase A
n: number of participants for a given subset of the sample
N: total number of participants in the sample
OFC/VLPFC: orbitofrontal plus ventrolateral prefrontal cortex
SD: standard deviation
vmPFC: ventromedial prefrontal cortex.
