# Supplementary material for: Neuroscientific and Genetic Evidence in Criminal Cases: A Double-Edged Sword in Germany but Not in the United States?
Source: Front Psychol. 2019 Oct 16;10:2343. doi: 10.3389/fpsyg.2019.02343 (PMC6805698; doi:10.3389/fpsyg.2019.02343)
Supplement: Supplementary file 1 [file Table_1.DOCX]

**Supplementary Material**

**Case Vignettes**

**Case**

Stefan (age 22 at the time) entered a drug store in the evening. When he saw Sandra (also 22 years old at the time), he demanded for explanations for the accusations made by her to the police about him. Stefan started to argue with her, and suddenly he took out a kitchen knife and stabbed Sandra 4 times. She died in the drugstore. According to investigators, Sandra had broken up the love relationship with Stefan of about one year several weeks ago, because he had been aggressive and abusive. Sandra had filed several complaints about his threats. Stefan eventually was arrested and convicted of manslaughter. That Stefan had committed the manslaughter was beyond any reasonable doubt.

**Background Information**

The German law provides for *manslaughter* at least 5 years, and in especially severe cases a life sentence (German Penal Code, § 212).

Mentally disordered convicts can either serve their sentence in a prison or a forensic psychiatric hospital. This depends on whether the disorder had impacted the capacity to understand the wrongfulness the act or the ability to control conduct during the offence, and thus the criminal responsibility.

Convicts who have been declared criminally responsible despite their disorder are imprisoned. On the other hand, those who are found to be legally irresponsible (German Penal Code, § 20) or sentenced with ‘diminished responsibility’ (German Penal Code, § 21) due to a psychiatric disorder are sent to a forensic psychiatric hospital (German Penal Code, § 63) if they are considered to pose a threat to society.

Please assume now the role of the judge presiding over Stefan’s sentencing hearing. Assume that all testimonies and related facts below were examined in court and proven beyond any reasonable doubt.

All groups received the following description:

**Diagnosis of Psychopathy**

Dr. Pichet, a psychiatrist and renowned expert of psychopathy, is heart in court. The expert diagnosed Stefan with psychopathy. Psychopathy itself is not currently included in the Diagnostic and Statistical Manual (DSM), a tool for making psychiatric diagnoses. However, there is a talk of it being in the next version of the DSM, since psychopathy is recognized as a unique subset of antisocial personality disorder, a disorder which is in the DSM. The diagnosis of psychopathy is defined by impulsivity; irresponsibility; shallow emotions; lack of empathy, guilt, or remorse; pathological lying; manipulation; superficial charm; and the persistent violation of social norms and expectations. Trained clinicians diagnose psychopathy with the validated diagnostic tool Psychopathy Checklist-Revised (PCL-R).

Dr. Pichet continued that psychopaths’ antisocial behavior follows from a broken emotional system: Psychopaths are without conscience; they have no empathy toward other people. Psychopathy results from dysfunctional moral socialization. Moral socialization is the process whereby humans (from childhood, through adolescence, and into adulthood) learn what is right and wrong. Normal children, when they inflict harm on someone else, will recognize the distress that they have caused someone else and so they can be taught that such behavior is inappropriate. But psychopaths are resistant to moral socialization because of their disorder. They do not understand what is right and wrong as the rest of us do. This may be why they are so resistant to treatment.

The second group received additionally the following explanation:

**Additional Neurological Diagnosis: Brain injury**

Then Dr. Marell was heart by the court. He is a neurologist and renowned expert of acquired psychopathy. He had performed an investigation of Stefan’s brain with Magnetic Resonance Imaging (MRI), and had conducted clinical interviews with Stefan’s family.

Dr. Marell concluded that a key player was Stefan’s severe car accident in which he obtained a traumatic brain injury. The interviews with family indicated that after the serious car crash Stefan “was not the same”. “He became more violent, he had sudden changes in emotion, it´s like he was not Stefan anymore, he was a completely different person after that accident”, his family stated.

Dr. Marell explains that traumatic brain injury, especially injuries in the frontal lobe, can be a cause of psychopathy. Dr. Marell argued that Stefan’s behavioral changes and personality changes were the direct result of the damage to the frontal lobe. This damage had caused deficits in rational decision-making and emotion processing. Dr. Marell mentions a similar, very popular case of the 1860´s, in which Phineas Gage, a 25-year-old man, had an accident at his workplace that resulted in an iron bar passing through the left side of his skull. Despite extensive damage to his forehead, he survived. According to Gage´s doctor, he became “fitful, irreverent, manifesting little deference for his fellows, impatient of restraint or advice when it conflicts with his desires”. Dr. Marell drew similarities between Gage´s and Stefan´s case.

Dr. Marell notes that Sandra filed several accusations about Stefan´s violent behavior only after he had had the car accident. Dr. Marell concludes that Stefan’s psychopathy has been caused by the brain trauma.

The third group received the following explanation:

**Additional Neurological Diagnosis: Low MAOA gene activity**

Then Dr. Adler is heart by the court. Dr. Adler is a geneticist and renowned expert for the genetics of psychopathy. Dr. Adler explained that recent peer-reviewed publications reported that the MAOA gene was associated with antisocial behavior. He explained that some humans have the gene for high MAOA activity, while others have the gene for low MAOA activity. Individuals with low MAOA activity were more likely to engage in antisocial behavior.

The reason for that is, according to Dr. Adler, that low MAOA activity has a detrimental effect on brain development, especially on the development of the amygdala. The amygdala is a brain structure involved in emotional processing and learning.

Extensive research had shown that normal humans have a violence-inhibition mechanism. This mechanism is controlled largely by the amygdala. It automatically causes anxiety in normal humans when they recognize that other humans are in pain or distress.

Psychopaths, Dr. Adler explained, have genetically-induced dysfunctional violence-inhibition mechanism. Therefore, they simply do not have the biological resources to experience anxiety in the face of others’ suffering. Consequently, they do not learn to associate distress in others with anxiety in themselves and are thus resistant to moral socialization.

Dr. Adler reported he had genetically tested Stefan for his MAOA status and had found that Stefan's genes confer low MAOA activity.

And the following conclusion will be provided at the end for all groups:

The prosecution requested that Stefan´s diagnosis of psychopathy should be considered an aggravating circumstance, since he poses a threat to society. The defense requested that Stefan’s diagnosis of psychopathy should be considered a mitigating circumstance, since Stefan has great difficulties to control his impulses due psychopathy.

**Questionnaire**

1. Please rate the degree of Stefan’s moral responsibility on a scale from 1 to 5 (1= no moral responsibility at all; to: 5 = completely morally responsible).

________

1. Please rate to which degree Stefan had a free will at the time of the manslaughter (in comparison with ordinary people) on a scale from 1 to 5 (from 1= no free will at all; 5 = complete free will).

________

1. Please rate Stefan’s legal responsibility:

- no legal responsibility
- diminished legal responsibility
- full legal responsibility

1. Would you send Stefan to prison or to a forensic psychiatric hospital?

- Prison
- Forensic psychiatric hospital
- No answer

1. In case that you decided to send Stefan to prison: Please provide a sentence.

________ years

1. Please indicate on a 5-point scale in how far the expert’s testimony affected your decision about whether to send Stefan to prison or to a forensic hospital (1= greatly mitigates, 3 = no effect, 5= greatly aggravates).

________

1. In case that you decided to send Stefan to prison: Please indicate on a 5-point scale in how far the expert’s testimony affected your decision about the number of years Stefan has to stay in prison (1= greatly mitigates, 3 = no effect, 5= greatly aggravates).

________

**For statistical analysis, please answer a few questions about yourself.**

1. Your gender

- Male
- Female
- No answer/other

1. Your semester of legal studies: ________
2. Your highest level of biological science training

- School until class 10
- Until university-entrance diploma
- Study of biology or medicine (not completed)
- Study of biology or medicine (completed)
- No answer

1. Your knowledge about psychopathy is influenced by: (several answers possible)

- Movies
- Fictional literature
- School
- Popular science magazines
- TV documentations
- Scientific articles and/or scientific books
- Nothing at all

**Statistical analysis**

**(S1) Moral responsibility**

*Fixed-Effects* ANOVA indicate a trend-level significant differences between the groups (*F*(2, 294) = 13.07, *p* < .006, $\eta_{p}^{2}$ = .03). The partial η^2^ =.03 and 90% CI suggest that this effect is of small to possibly moderate effect size. Only trend-level significant main effect. No post hoc t-test calculated.

Table S1 *Fixed-Effects ANOVA results using as.numeric(outc.moralresp) as the criterion*

| Predictor | Sum  of  Squares | *df* | Mean  Square | *F* | *p* | _partial_ η^2^ | _partial_ η^2^  90% CI  [LL, UL] |
| --- | --- | --- | --- | --- | --- | --- | --- |
| (Intercept) | 51.76 | 1 | 51.76 | 40.77 | .000 |  |  |
| Groups | 13.07 | 2 | 6.54 | 5.15 | **.006** | .03 | [.01, .07] |
| as.factor(covar.gender) | 2.00 | 1 | 2.00 | 1.58 | .210 | .01 | [.00, .03] |
| as.factor(covar.n_semesters) | 7.16 | 9 | 0.80 | 0.63 | .774 | .02 | [.00, .02] |
| as.factor(covar.biology_training) | 10.43 | 2 | 5.21 | 4.11 | .017 | .03 | [.00, .06] |
| as.factor(covar.psychopathy_knowledge) | 4.08 | 6 | 0.68 | 0.54 | .781 | .01 | [.00, .01] |
| as.factor(covar.university) | 2.05 | 2 | 1.02 | 0.81 | .447 | .01 | [.00, .02] |
| Error | 373.22 | 294 | 1.27 |  |  |  |  |

*Note.* LL and UL represent the lower-limit and upper-limit of the partial η^2^ confidence interval, respectively.


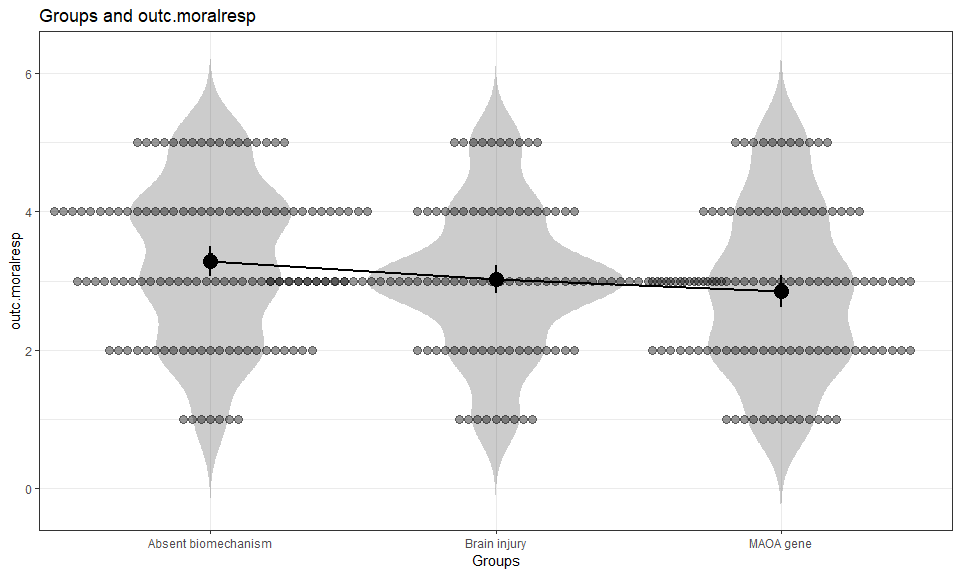


Figure S1 Violin plot visualize the distribution of the data and its probability density. The black dot indicates the group mean and the width of the gray area displays the frequency of the category assigned (1 = “Not at all”, 2 = “Diminished”, 3 = “Fully”). The mean answer in the “Absent Biomechanism” group is 3.29 (SD = 1.14) and in the “MAOA gene” group 2.85 (SD = 1.19).

**(S2) Free will**

No significant main effect. No post hoc t-test calculated.

Table S2 *Fixed-Effects ANOVA results using as.numeric(outc.freewill) as the criterion*

| Predictor | Sum  of  Squares | *df* | Mean  Square | *F* | *p* | _partial_ η^2^ | _partial_ η^2^  90% CI  [LL, UL] |
| --- | --- | --- | --- | --- | --- | --- | --- |
| (Intercept) | 66.58 | 1 | 66.58 | 58.03 | .000 |  |  |
| Groups | 9.07 | 2 | 4.54 | 3.95 | .020 | .03 | [.00, .06] |
| as.factor(covar.gender) | 2.75 | 1 | 2.75 | 2.40 | .123 | .01 | [.00, .03] |
| as.factor(covar.n_semesters) | 13.66 | 9 | 1.52 | 1.32 | .224 | .04 | [.00, .05] |
| as.factor(covar.biology_training) | 0.13 | 2 | 0.06 | 0.06 | .945 | .00 | [.00, .00] |
| as.factor(covar.psychopathy_knowledge) | 9.93 | 6 | 1.66 | 1.44 | .198 | .03 | [.00, .05] |
| as.factor(covar.university) | 1.50 | 2 | 0.75 | 0.66 | .520 | .00 | [.00, .02] |
| Error | 337.35 | 294 | 1.15 |  |  |  |  |

*Note.* LL and UL represent the lower-limit and upper-limit of the partial η^2^ confidence interval, respectively.

**(S3) Legal responsibility**

*Fixed-Effects* ANOVA indicate a significant differences between the groups (*F*(2, 294) = 4.80, *p* < .001, $\eta_{p}^{2}$ = .05). The partial η^2^ =.05 and 90% CI suggest that this effect is of small to possibly moderate effect size. Post hoc t-tests revealed significant differences between the group that received no biological explanation and the “brain injury” group (t(213.04) = 3.27, p = .004), i.e. the group that received no biological explanation assigned a higher legal responsibility.

Table S3 *Fixed-Effects ANOVA results using as.numeric(outc.legalresp) as the criterion*

| Predictor | Sum  of  Squares | *df* | Mean  Square | *F* | *p* | _partial_ η^2^ | _partial_ η^2^  90% CI  [LL, UL] |
| --- | --- | --- | --- | --- | --- | --- | --- |
| (Intercept) | 23.32 | 1 | 23.32 | 79.97 | .000 |  |  |
| Groups | 4.80 | 2 | 2.40 | 8.24 | **.000** | .05 | [.02, .10] |
| as.factor(covar.gender) | 1.00 | 1 | 1.00 | 3.44 | .065 | .01 | [.00, .04] |
| as.factor(covar.n_semesters) | 2.62 | 9 | 0.29 | 1.00 | .441 | .03 | [.00, .04] |
| as.factor(covar.biology_training) | 1.03 | 2 | 0.52 | 1.76 | .174 | .01 | [.00, .04] |
| as.factor(covar.psychopathy_knowledge) | 2.50 | 6 | 0.42 | 1.43 | .204 | .03 | [.00, .05] |
| as.factor(covar.university) | 1.04 | 2 | 0.52 | 1.79 | .169 | .01 | [.00, .04] |
| Error | 85.72 | 294 | 0.29 |  |  |  |  |

*Note.* LL and UL represent the lower-limit and upper-limit of the partial η^2^ confidence interval, respectively.


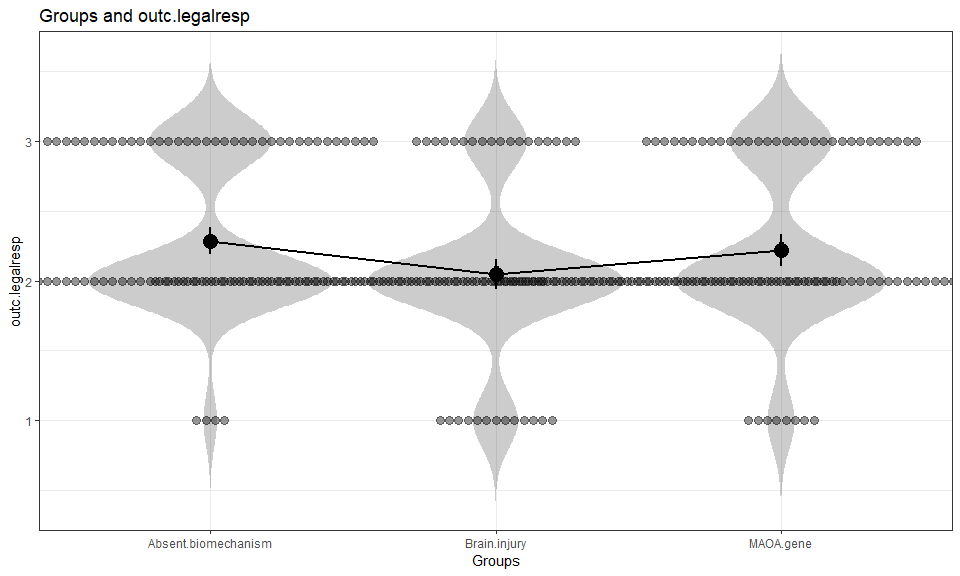


Figure S2 Violin plot visualize the distribution of the data and its probability density. The black dot indicates the group mean and the width of the gray area displays the frequency of the category assigned (1 = “Not at all”, 2 = “Diminished”, 3 = “Fully”). The mean answer in the “absent biomechanism” group is 2.29 (SD=0.53) and in the “brain injury” group 2.05 (SD=0.54).

**(S4) Type of custody assigned (prison versus forensic hospital)**

No significant main effect. No post hoc t-test calculated.

Table S4 *Fixed-Effects ANOVA results using as.numeric(outc.custody) as the criterion*

| Predictor | Sum  of  Squares | *df* | Mean  Square | *F* | *p* | _partial_ η^2^ | _partial_ η^2^  90% CI  [LL, UL] |
| --- | --- | --- | --- | --- | --- | --- | --- |
| (Intercept) | 28.18 | 1 | 28.18 | 124.15 | .000 |  |  |
| Groups | 1.87 | 2 | 0.94 | 4.12 | .017 | .03 | [.00, .06] |
| covar.gender | 0.02 | 1 | 0.02 | 0.08 | .780 | .00 | [.00, .01] |
| as.numeric(covar.n_semesters) | 0.11 | 1 | 0.11 | 0.49 | .485 | .00 | [.00, .02] |
| as.ordered(covar.biology_training) | 0.31 | 2 | 0.15 | 0.69 | .502 | .00 | [.00, .02] |
| as.ordered(covar.psychopathy_knowledge) | 1.96 | 6 | 0.33 | 1.44 | .198 | .03 | [.00, .05] |
| covar.university | 2.44 | 2 | 1.22 | 5.37 | .005 | .03 | [.01, .07] |
| Error | 68.56 | 302 | 0.23 |  |  |  |  |

*Note.* LL and UL represent the lower-limit and upper-limit of the partial η^2^ confidence interval, respectively.

**(S5) Influence on the type of custody assigned**

No significant main effect. No post hoc t-test calculated.

Table S5 *Fixed-Effects ANOVA results using as.numeric(outc.influenceCustody) as the criterion*

| Predictor | Sum  of  Squares | *df* | Mean  Square | *F* | *p* | _partial_ η^2^ | _partial_ η^2^  90% CI  [LL, UL] |
| --- | --- | --- | --- | --- | --- | --- | --- |
| (Intercept) | 81.53 | 1 | 81.53 | 42.75 | .000 |  |  |
| Groups | 1.45 | 2 | 0.72 | 0.38 | .685 | .00 | [.00, .01] |
| covar.gender | 0.18 | 1 | 0.18 | 0.10 | .758 | .00 | [.00, .01] |
| as.numeric(covar.n_semesters) | 1.22 | 1 | 1.22 | 0.64 | .424 | .00 | [.00, .02] |
| as.ordered(covar.biology_training) | 10.94 | 2 | 5.47 | 2.87 | .058 | .02 | [.00, .05] |
| as.ordered(covar.psychopathy_knowledge) | 8.23 | 6 | 1.37 | 0.72 | .635 | .01 | [.00, .02] |
| covar.university | 4.15 | 2 | 2.08 | 1.09 | .338 | .01 | [.00, .03] |
| Error | 576.02 | 302 | 1.91 |  |  |  |  |

*Note.* LL and UL represent the lower-limit and upper-limit of the partial η^2^ confidence interval, respectively.

**(S6) Duration of sentencing assigned**

No significant main effect. No post hoc t-test calculated.

Table S6 *Fixed-Effects ANOVA results using as.numeric(outc.sentencing) as the criterion*

| Predictor | Sum  of  Squares | *df* | Mean  Square | *F* | *p* | _partial_ η^2^ | _partial_ η^2^  90% CI  [LL, UL] |
| --- | --- | --- | --- | --- | --- | --- | --- |
| (Intercept) | 177.22 | 1 | 177.22 | 9.47 | .003 |  |  |
| Groups | 23.88 | 2 | 11.94 | 0.64 | .530 | .01 | [.00, .05] |
| covar.gender | 3.19 | 1 | 3.19 | 0.17 | .681 | .00 | [.00, .04] |
| as.numeric(covar.n_semesters) | 1.04 | 1 | 1.04 | 0.06 | .814 | .00 | [.00, .02] |
| as.ordered(covar.biology_training) | 1.13 | 2 | 0.56 | 0.03 | .970 | .00 | [.00, 1.00] |
| as.ordered(covar.psychopathy_knowledge) | 41.78 | 6 | 6.96 | 0.37 | .895 | .02 | [.00, .02] |
| covar.university | 1.15 | 2 | 0.57 | 0.03 | .970 | .00 | [.00, 1.00] |
| Error | 1984.18 | 106 | 18.72 |  |  |  |  |

*Note.* LL and UL represent the lower-limit and upper-limit of the partial η^2^ confidence interval, respectively.

**(S7) Influence on the duration of sentencing assigned**

No significant main effect. No post hoc t-test calculated.

Table S7 *Fixed-Effects ANOVA results using as.numeric(outc.influenceSentence) as the criterion*

| Predictor | Sum  of  Squares | *df* | Mean  Square | *F* | *p* | _partial_ η^2^ | _partial_ η^2^  90% CI  [LL, UL] |
| --- | --- | --- | --- | --- | --- | --- | --- |
| (Intercept) | 24.22 | 1 | 24.22 | 14.34 | .000 |  |  |
| Groups | 2.32 | 2 | 1.16 | 0.69 | .505 | .01 | [.00, .06] |
| covar.gender | 7.18 | 1 | 7.18 | 4.25 | .042 | .04 | [.00, .11] |
| as.numeric(covar.n_semesters) | 1.53 | 1 | 1.53 | 0.91 | .343 | .01 | [.00, .06] |
| as.ordered(covar.biology_training) | 3.79 | 2 | 1.90 | 1.12 | .329 | .02 | [.00, .07] |
| as.ordered(covar.psychopathy_knowledge) | 6.52 | 6 | 1.09 | 0.64 | .695 | .04 | [.00, .05] |
| covar.university | 5.95 | 2 | 2.98 | 1.76 | .177 | .03 | [.00, .09] |
| Error | 177.34 | 105 | 1.69 |  |  |  |  |

*Note.* LL and UL represent the lower-limit and upper-limit of the partial η^2^ confidence interval, respectively.
